# Lowering the Sampling Rate: Heart Rate Response during Cognitive Fatigue

**DOI:** 10.3390/bios12050315

**Published:** 2022-05-10

**Authors:** Kar Fye Alvin Lee, Elliot Chan, Josip Car, Woon-Seng Gan, Georgios Christopoulos

**Affiliations:** 1 Nanyang Business School, Nanyang Technological University, Singapore 639798, Singapore; alvin.lee@ntu.edu.sg (K.F.A.L.); elliotchan@ntu.edu.sg (E.C.); 2 Lee Kong Chian School of Medicine, Nanyang Technological University, Singapore 308232, Singapore; josip.car@ntu.edu.sg; 3 Smart Nation Translational Laboratory, School of Electrical and Electronic Engineering, Nanyang Technological University, Singapore 639798, Singapore; ewsgan@ntu.edu.sg

**Keywords:** cognitive fatigue, heart rate variability, biomarker, sampling rate

## Abstract

Cognitive fatigue is a mental state characterised by feelings of tiredness and impaired cognitive functioning due to sustained cognitive demands. Frequency-domain heart rate variability (HRV) features have been found to vary as a function of cognitive fatigue. However, it has yet to be determined whether HRV features derived from electrocardiogram data with a low sampling rate would remain sensitive to cognitive fatigue. Bridging this research gap is important as it has substantial implications for designing more energy-efficient and less memory-hungry wearables to monitor cognitive fatigue. This study aimed to examine (1) the level of agreement between frequency-domain HRV features derived from lower and higher sampling rates, and (2) whether frequency-domain HRV features derived from lower sampling rates could predict cognitive fatigue. Participants (*N* = 53) were put through a cognitively fatiguing 2-back task for 20 min whilst their electrocardiograms were recorded. Results revealed that frequency-domain HRV features derived from sampling rate as low as 125 Hz remained almost perfectly in agreement with features derived from the original sampling rate at 2000 Hz. Furthermore, frequency domain features, such as normalised low-frequency power, normalised high-frequency power, and the ratio of low- to high-frequency power varied as a function of increasing cognitive fatigue during the task across all sampling rates. In conclusion, it appears that sampling at 125 Hz is more than adequate for frequency-domain feature extraction to index cognitive fatigue. These findings have significant implications for the design of low-cost wearables for detecting cognitive fatigue.

## 1. Introduction

Cognitive fatigue is a mental state characterised by the subjective feelings of tiredness, having difficulty focusing, and/or an impaired ability to think [1,2]. This mental state is a result of cognitive “overloading” by engaging in cognitively demanding activities for a prolonged period of time [1,3,4,5,6,7]. Previous research has found that cognitive fatigue was associated with negative effects on cognitive functioning, such as working memory, judgement, and attention [8,9,10,11]. Hence, cognitive fatigue may result in more mistakes and, consequentially, the risk of accidents [12,13]. Hence, advancing assessment methods of cognitive fatigue is imperative in managing such risk, especially in critical situations.

A recent review has suggested that heart rate variability (HRV) is a potential biomarker of cognitive fatigue [14]. In particular, the review highlighted multiple studies demonstrating that HRV features extracted through frequency-domain analysis, such as high-frequency (HF) power, low-frequency (LF) power, and the ratio of low- to high-frequency (LF/HF) power, varied as a function of cognitive fatigue. For instance, previous research has demonstrated a decrease in HF during, as well as before and after, prolonged moderately demanding cognitive tasks [15,16,17,18]. By contrast, these studies also observed an increase in LF. Notably, HF has been hypothesised to reflect parasympathetic nervous activity, whereas LF has been hypothesised to reflect both sympathetic and parasympathetic nervous activities [19,20,21,22,23]. Some researchers have further proposed LF/HF as a better representation of the interplay between the sympathetic and parasympathetic nervous systems as compared to LF or HF individually [20,24]. Indeed, some studies have demonstrated that LF/HF increased during and after engaging in cognitively demanding tasks [17,18]. Overall, there is empirical evidence indicating that both the sympathetic and parasympathetic nervous systems are differentially involved in cognitive fatigue. Specifically, it appears that a shift towards sympathetic predominance in the sympathovagal balance may be linked to cognitive fatigue. 

Given the advancement in wearable technology with the capability to record various physiological measures, such as heart rate [25], the potential of continuously monitoring an individual’s cognitive fatigue levels beyond the laboratory appears to be viable. However, unlike laboratory-based electrocardiogram (ECG) recording devices, which predominant consideration is accuracy, commercially available wearables have other considerations, such as cost, form factor, memory, computation time, and battery life [26]. Taking into account these considerations, wearables often compromise on sampling rate as compared to their laboratory counterparts. For instance, Polar H10 (https://www.polar.com/sg-en/products/accessories/h10_heart_rate_sensor/; accessed on 25 March 2022) is a typical example of a commercially available wearable and has a sampling rate of 130 Hz, whereas Biopac BN-RSPEC (https://www.biopac.com/product/bionomadix-rsp-with-ecg-amplifier/; accessed on 25 March 2022), an example of a laboratory-based device, samples at 2000 Hz. Notably, a lower sampling rate may cause jittering in the R-peaks, introducing greater measurement error during peak detection and, consequentially, affecting the derivations of HRV features [27]. Indeed, previous research using simulated ECG data demonstrated that decreased sampling rate has a negative influence on the signal-to-noise ratio of the HRV frequency-domains [28]. The minimum sampling rate for frequency-domain analysis recommended by various researchers ranged from 100 Hz to 250 Hz [29,30,31]. Although the focus of this study pertained to non-adaptive offline processing, it should be noted that employing more sophisticated processing methods, such as adaptive filtering [32], may require a higher sampling rate. Overall, it appears that the minimum sampling rate required for frequency-domain HRV feature extraction, particularly in the context of using these features to predict cognitive fatigue, remains to be investigated.

This study aimed to better understand whether frequency-domain HRV features derived from lower sampling rate ECG data could predict cognitive fatigue. To this end, this study collected ECG data sampled at 2000 Hz during a typical cognitively fatiguing task via a laboratory-based ECG recording device, systematically down-sampled the ECG data, extracted frequency-domain features from the ECG data of different sampling rates, and build predictive models based on these features.

## 2. Materials and Methods

### 2.1. Participants

Ethics approval was obtained from Nanyang Technological University’s Institutional Review Board (IRB-2020-10-041). The study was advertised online through research invitation-themed group chat, SONA, and clinicaltrial.gov, and offline through flyers posted within Nanyang Technological University. Data collection was conducted as part of a larger study examining the effects of cognitive fatigue on HRV and skin conductance (Clinical Trials Identifier: NCT05011318; https://clinicaltrials.gov/ct2/show/NCT05011318; accessed on 25 March 2022). Informed consent was obtained from all participants before the start of the experiment. The sample consisted of 53 young adults (*M* = 22.91, *SD* = 2.13), 30 of whom were females.

### 2.2. Cognitive Fatiguing Task

**Figure 1:**
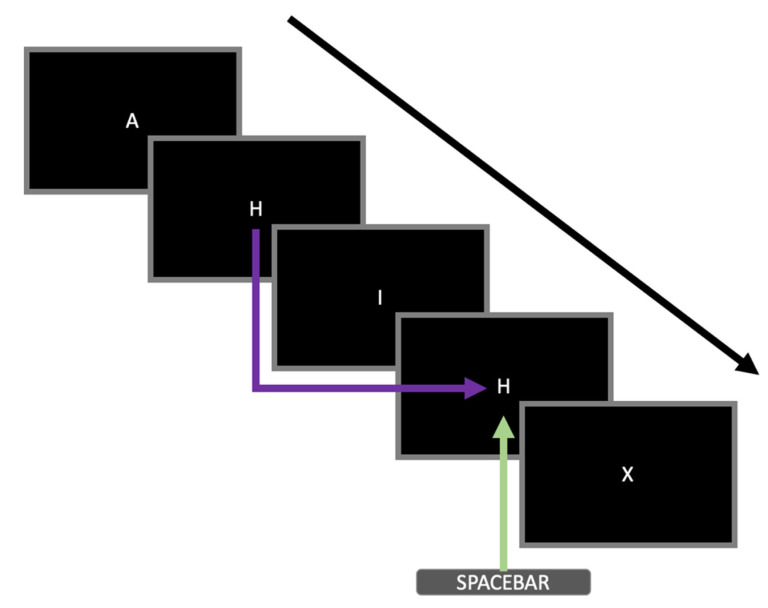
An example of a sequence of stimuli presented during the 2-back task adopted in this study. Five trials are shown in this figure. The black arrow represents the temporal sequence of the letters presented during the task. The purple arrow highlights that the particular stimulus was identical to the stimulus two trials back (i.e., matching trial). The green arrow indicates that the participant had to respond by pressing the spacebar during that particular trial.

An adapted version of the *N*-back task [33] was administered to invoke cognitive fatigue in participants to measure their heart rate responses. Only the 2-back condition was administered. The stimulus consisted of white letters (A, H, I, X) presented at the centre of the screen. A sequence of letters was presented to the participants. Only a single letter appeared on the screen at any one time. Participants were required to respond by pressing the spacebar when the current letter was identical to the letter presented two positions back (i.e., matching trial, see Figure 1 for an example). Each stimulus was presented for 500 ms. The intertrial interval was 2500 ms. Trial-to-trial feedback was not provided. Participants were instructed to respond as fast and as accurately as they could. Eight practice trials, which consisted of two matching trials, were administered at the start to ensure that the participants understood the instructions. The actual task consisted of four blocks of 100 trials. In each block, 25 trials were matching trials. The order of the trials was randomised. The total number of trials was selected based on previous research by Tanaka et al. [34], which used a similar task, demonstrating that at least 15 min were required for the onset of cognitive fatigue to be observable physiologically. Our task consisted of a total of 400 trials, which lasted for 20 min.

### 2.3. Electrocardiogram Data Acquisition and Processing

Continuous single-lead ECG was recorded using Biopac^TM^ MP160 with RSPEC-R module. The ECG electrodes were positioned in the lead-II position with the negative electrode on the right clavicle, the positive electrode on the lower left rib, and the ground electrode on the lower right rib (refer to the following link for a diagram; https://www.biopac.com/wp-content/uploads/ECG-Guide.pdf; accessed on 25 March 2022). The analogue-to-digital conversion was set at 16-bit resolution with a sampling rate of 2000 Hz. An online bandpass filter (1–35 Hz) was applied during recording. 

**Figure 2:**
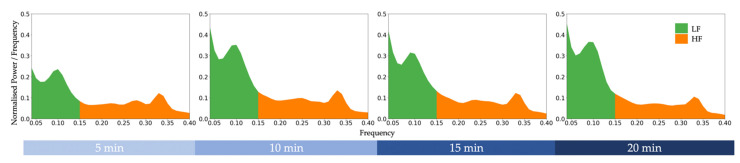
Grand averaged power spectral density graph of the original data (sampled at 2000 Hz) across all participants during a cognitively fatiguing task. LF = low-frequency power. HF = high-frequency power.

ECG data were processed using Neurokit2 [35]. The recordings were first down-sampled using decimation from 2000 Hz to 1000 Hz, 500 Hz, 250 Hz, and 125 Hz. That is, every *M^th^* sample was kept with respect to their target sampling rate. The different sampling rate datasets were processed independently. Each dataset was digitally filtered using the default Neurokit2 clean function to remove line noise. The default peak detection algorithm from Neurokit2 was used to identify R-peaks. R-peaks affected by abnormal ECG and artefacts were replaced by mid-point peak insertions derived from adjacent peaks. Each block of 100 trials was extracted into a total of four 5-min blocks. Frequency-domain HRV features were calculated for each block via Welch’s method [36]. The grand averaged power spectral density graph of the original data across all participants is reported in Figure 2.

## 3. Results

The descriptive statistics for the frequency-domain HRV features across all sampling rates are reported in Table 1. 

**Figure 3:**
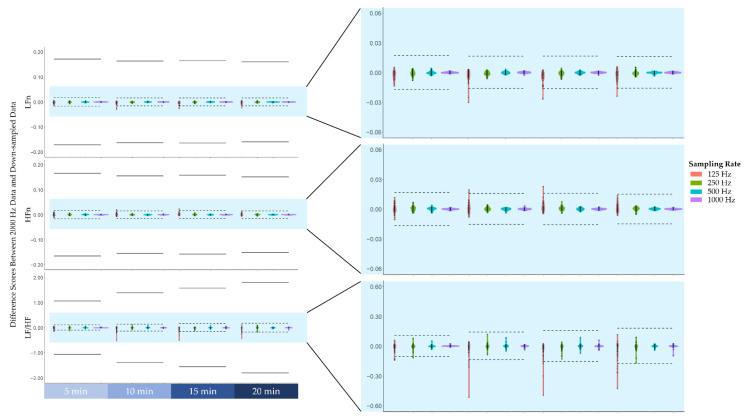
The difference scores between the frequency-domain heart rate variability features derived from the original 2000 Hz data and features derived from down-sampled data (i.e., 1000 Hz, 500 Hz, 250 Hz, 125 Hz). From top to bottom, difference scores of low-frequency power normalised to total power (LFn), high-frequency power normalised to total power (HFn), and the ratio of low-frequency power to high-frequency power (LF/HF). Note that the solid lines represent ± 1 standard deviation of the features derived from the 2000 Hz data, whereas the dashed lines represent ± 1/10 standard deviation.

Prior to examining the agreement levels between frequency-domain HRV features across the different sampling rates, the difference scores between the features derived from the original data (2000 Hz) and features derived from down-sampled data (1000 Hz, 500 Hz, 250 Hz, 125 Hz) across the four blocks are plotted in Figure 3. As can be seen in Figure 3, the difference scores did not exceed 1 standard deviation of the 2000 Hz features.

**Figure 4:**
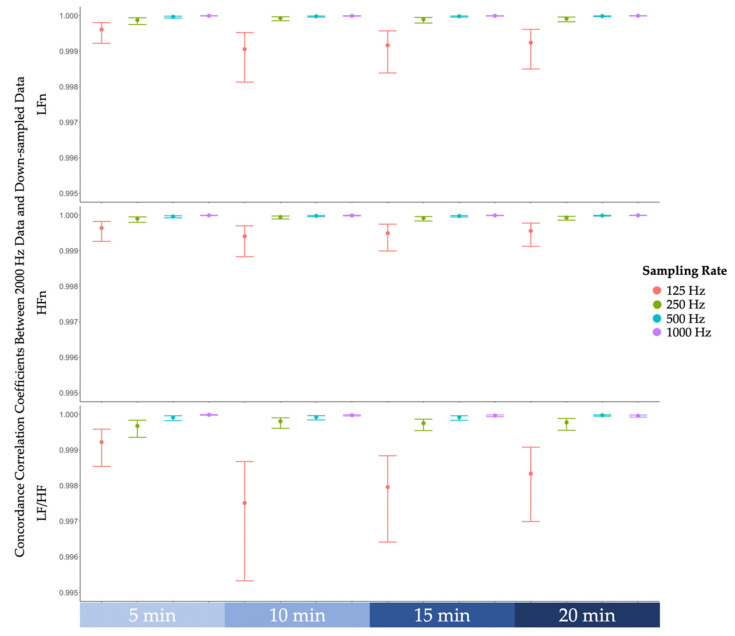
The concordance correlation coefficients between frequency-domain features derived from the original 2000 Hz data and features derived from down-sampled data (i.e., 1000 Hz, 500 Hz, 250 Hz, 125 Hz). From top to bottom, concordance correlation coefficients of low-frequency power normalised (LFn), high-frequency power normalised (HFn), and the ratio of low-frequency power to high-frequency power (LF/HF). Note that the error bars represent 99% confidence intervals.

Lin’s concordance correlation coefficients between features derived from the original data sampled at 2000 Hz and features derived from down-sampled data were examined [37]. As reported in Figure 4, despite a drop in concordance correlation coefficients at 125 Hz, all coefficients were greater than 0.99, indicating that the strength of agreement between 2000 Hz and lower sampling rates is almost perfect [38].

**Figure 5:**
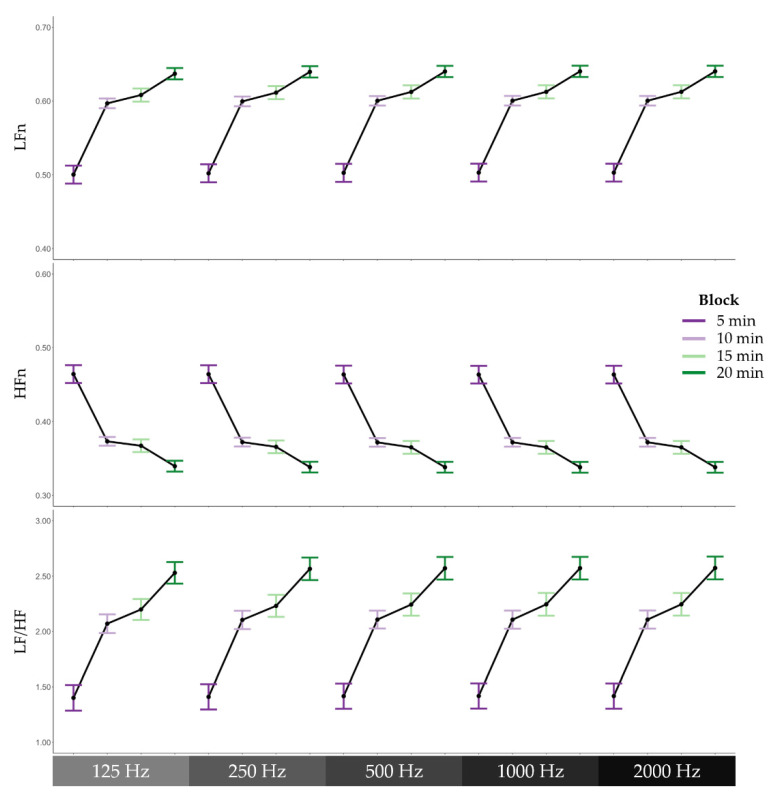
Frequency domain heart rate variability features across 5-min blocks during the cognitively fatiguing task. From top to bottom, means and standard errors of low-frequency power normalised to total power (LFn), high-frequency power normalised to total power (HFn), and the ratio of low-frequency power to high-frequency power (LF/HF), and from left to right, the different sampling rates, 125 Hz, 250 Hz, 500 Hz, 1000 Hz, and 2000 Hz.

To examine whether HRV features derived from different sampling rates varied as a function of cognitive fatigue, three regression models were conducted with block and sampling rate as predictors and low-frequency power normalised (LFn), high-frequency power normalised (HFn), or the ratio of low-frequency power to high-frequency power (LF/HF) as the outcome variable of each model. The Satterthwaite’s method was used for degrees of freedom estimation [39]. With LFn as the outcome variable, the regression model revealed a significant effect of block, *F*(1, 1004) = 217.60, *p* < 0.001. As can be seen in Figure 5, LFn increased across the four blocks during the 2-Back task. There was no significant effect of sampling rate, *F*(1, 1004) = 0.02, *p* = 0.892. In addition, there was no significant interaction effect between block and sampling rate on LFn, *F*(1, 1004) = 0.00, *p* = 0.982. In the context of the HFn model, the regression model revealed a significant effect of block, *F*(1, 1004) = 190.37, *p* < 0.001. As illustrated in Figure 5, HFn decreased across the four blocks during the task. There was no significant effect of sampling rate, *F*(1, 1004) = 0.00, *p* = 0.969, nor was there a significant interaction effect, *F*(1, 1004) = 0.00, *p* = 0.977. In the LF/HF model, there was also a significant effect of block, *F*(1, 1004) = 135.71, *p* < 0.001. Again, there was no significant effect of sampling rate, *F*(1, 1004) = 0.00, *p* = 0.953, nor interaction effect, *F*(1, 1004) = 0.01, *p* = 0.923.

## 4. Discussion

This study aimed to examine whether frequency-domain HRV features derived from lower sampling rate ECG data could predict cognitive fatigue. We first examined the difference scores between the frequency-domain HRV features derived from lower sampling rate data and the original data, which was sampled at 2000 Hz. We found that the majority of the difference scores remained within a tenth of the standard deviation of the frequency-domain HRV features derived from the original data. Only some difference scores at lower sampling rates exceeded a tenth of the standard deviation but remained well within one standard deviation. On this basis, there appears to only be a slight deviation between frequency-domain HRV features derived from lower sampling rate data and the original data. Further analysis corroborated this notion by demonstrating that the concordance correlation coefficients between frequency-domain HRV features derived from lower sampling rate data and the original data were all above 0.99. According to the guidelines stipulated by McBride [38], our results indicated that the strength of agreement was almost perfect. Finally, the predictive models based on the frequency-domain HRV features derived across all sampling rates varied as a function of cognitive fatigue. In particular, low-frequency power was found to increase over time during the cognitively fatiguing task, whereas high-frequency power was found to decrease over time during the task. Furthermore, the ratio of low-frequency power to high-frequency power also increased over time. Our findings are consistent with previous research that observed a similar pattern of results [15,16,17,18]. Hence, there is empirical evidence supporting the notion that cognitive fatigue may shift the sympathovagal balance towards greater sympathetic predominance. Our findings further previous research by providing empirical evidence suggesting that a sampling rate of 125 Hz, which was previously contentious amongst researchers [29,30,31], is adequate for frequency-domain feature extraction to predict cognitive fatigue.

Given the discrepancy in recommended minimum sampling rate for frequency-domain analysis, it is important to compare the differences between our study and previous research. A key difference between our study and the study by Kwon and colleagues [30] was that they applied linear interpolation for down-sampling of the ECG data, while we utilised decimation for down-sampling. By contrast, the study by Ziemssen and colleagues [29] applied a rounding method to mimic the loss of temporal resolution post-peak detection. That is, the detected beat-to-beat interval was divided by a factor with respect to the target down-sampling rate, rounded to the nearest whole number, and then multiply by the factor again. Arguably, the method adopted by previous research does not adequately replicate the process of independently collecting ECG data with different sampling rates. Instead, both linear interpolation and post-peak detection rounding methodologies contain information beyond the targeted down-sampled rates. On the other hand, decimation prior to any post-processing of the ECG data removes any information beyond a given down-sampling rate, which is a more appropriate method for the purposes of our study. Nonetheless, future research should systematically examine how different down-sampling methodologies may affect frequency-domain HRV features. In addition, anti-aliasing filter was not applied intentionally as aliasing artefacts are a product of sampling rates below two times the Nyquist frequency [40,41,42,43,44], which was a non-issue even at our lowest down-sampling rate of 125 Hz. Although aliasing artefacts on the ECG signal may not be an issue at 125 Hz, it should be noted that the HRV signal is still susceptible to aliasing artefacts [45]. Hence, future researchers should explore viable algorithms to detect and remove aliasing artefacts in HRV signals. In any case, a potential reason for the inconsistency with previous research findings may be due to the different down-sampling methodologies adopted. We, however, argue that our methodology provides a better representation of the available information at each given sampling rate. Not surprisingly, a previous study, which adopted a similar down-sampling methodology to our study, suggested a similar minimum sampling rate [31]. It is important to note that our study collected ECG data while experimentally manipulating cognitive fatigue levels, whereas the study by Ellis et al. [31] used a subset of healthy controls from the Physikalisch-Technische Bundesanstalt Diagnostic ECG database [46], which did not involve any experimental manipulations.

Another key difference between our study and the previous research [29,30] was that we recruited healthy young adults from the general population. Kwon and colleagues, on the other hand, examined patients with acute cholinesterase inhibitor poisoning [30]. Indeed, cholinesterase inhibitors have been shown to have significant cardiovascular effects [47,48]. In comparison, Ziemssen and colleagues [29] recruited from both the clinical and general populations, which included healthy, diabetic, hypercholesterolemic, pregnant, hypertensive, and cardiac autonomic failure subjects (for more details regarding the Eurobarvar dataset, see http://www.eurobavar.altervista.org; accessed on 25 March 2022). By contrast, the study by Ellis and colleagues examined healthy subjects only [31], which may explain why our results are more consistent with their findings. Future studies would need to explore how abnormal heart conditions may moderate the minimum sampling rate required for frequency-domain feature extraction to predict cognitive fatigue. In addition to abnormal heart conditions, other factors, such as different age groups [49], should also be considered. Hence, our recommendation of 125 Hz should be limited to healthy young adults and should not be generalised to the clinical population. 

One of the key limitations of this study is that the ECG data were collected using a laboratory-based device only. Although we attempted to mimic the data quality of wearables through data down-sampling, future research should cross-validate the ECG data collected from wearables with laboratory equipment to examine whether wearables are suitable to predict cognitive fatigue. Another key limitation is that our ECG data were collected with non-moving participants (i.e., sitting in front of a computer). Given that one of the key advantages of wearables is mobility, future research should also examine how different levels of physical activity may moderate the minimum sampling rate required for frequency-domain feature extraction to predict cognitive fatigue. In addition, our data only consisted of healthy young adults, which certainly limit the generalisability of the models. Hence, future research should expand data collection to a broader age group to build more robust predictive models of cognitive fatigue. Despite these limitations, our study has provided empirical evidence indicating that ECG sampled at 125 Hz is more than adequate for frequency-domain feature extraction to predict cognitive fatigue in young adults during a computerised cognitive task. Notably, preliminary research has demonstrated that a reduction in sampling rate reduces energy consumption during real-time data transmission [50]. Hence, our findings pertaining to the adequacy of lower sampling rate have substantial implications for reducing the processing, battery, and memory demands of a wearable. Overall, our study is an important intermediary step towards the design of low-energy wearables that could be used to accurately monitor and predict cognitive fatigue.

## Figures and Tables

**Table 1:** Descriptive statistics of the frequency-domain heart rate variability features across different sampling rates.

HRV	Block 1 (5 min)		Block 2 (10 min)		Block 3 (15 min)		Block 4 (20 min)	
Features	*M*	*SD*	*M*	*SD*	*M*	*SD*	*M*	*SD*
**2000 Hz**								
LFn	0.50	0.17	0.60	0.16	0.61	0.16	0.64	0.16
HFn	0.46	0.17	0.37	0.15	0.36	0.16	0.34	0.15
LF/HF	1.42	1.06	2.11	1.39	2.24	1.56	2.57	1.80
**1000 Hz**								
LFn	0.50	0.17	0.60	0.16	0.61	0.16	0.64	0.16
HFn	0.46	0.17	0.37	0.15	0.36	0.16	0.34	0.15
LF/HF	1.42	1.06	2.11	1.39	2.24	1.57	2.57	1.79
**500 Hz**								
LFn	0.50	0.17	0.60	0.16	0.61	0.16	0.64	0.16
HFn	0.46	0.17	0.37	0.15	0.36	0.16	0.34	0.15
LF/HF	1.42	1.06	2.11	1.39	2.24	1.56	2.57	1.79
**250 Hz**								
LFn	0.50	0.17	0.60	0.16	0.61	0.16	0.64	0.16
HFn	0.46	0.17	0.37	0.15	0.37	0.16	0.34	0.15
LF/HF	1.41	1.05	2.10	1.40	2.23	1.55	2.57	1.79
**125 Hz**								
LFn	0.50	0.17	0.60	0.16	0.61	0.16	0.64	0.16
HFn	0.46	0.16	0.37	0.15	0.37	0.16	0.34	0.15
LF/HF	1.40	1.04	2.07	1.34	2.20	1.51	2.53	1.74

Note. *N* = 53. HRV = heart rate variability. LFn = low-frequency power (normalised to the total power). HFn = high-frequency power (normalised to the total power). LF/HF = the ratio of low-frequency to high-frequency power. Block 1 to 4 refers to each 5-min window across the 20-min 2-back task.

## Data Availability

Data are available upon reasonable request.
